# Symmetry in acute stroke imaging: a diagnostic pitfall

**DOI:** 10.1007/s00415-026-14250-w

**Published:** 2026-11-11

**Authors:** Eva S. Rickers, Lukas J. Volz, Gereon R. Fink, Lukas Hensel

**Affiliations:** 1 Faculty of Medicine, University of Cologne, and Department of Neurology, University Hospital Cologne, Cologne, Germany; 2 Cognitive Neuroscience, Institute of Neuroscience and Medicine (INM-3), Research Center Jülich, Jülich, Germany

Dear Sirs,

Acute stroke imaging is commonly interpreted with an emphasis on lateralized abnormalities. However, non-lateralizing deficits and bilateral symmetric lesions may also reflect acute vascular disease, and their recognition can have immediate implications for treatment. Here, we describe three patients who were admitted to the emergency department with atypical signs of acute stroke and were evaluated for recanalization therapy. All three showed symmetric bilateral cerebral imaging abnormalities, each reflecting a distinct pathophysiology. Through these cases, we discuss how recognizing symmetric bilateral lesions should alter acute management and reframe diagnostic reasoning, including when automated tools are used. Acknowledging lesion symmetry as a clinical and imaging ‘red flag’ may help to improve diagnostic accuracy and treatment in the hyperacute setting.

**Case 1.** A 64-year-old man presented with acute (< 2 h) neurologic deficits. Following singular pre-hospital emesis and transient aphasia, emergency room examination revealed mild right facial droop, pronator drift, psychomotor slowing, and cool extremities with weak, symmetric pulses. He had experienced mild, transient chest pain earlier on the day of admission and reported mild chronic lower back pain on presentation. Initial ECG was normal; heart rate was 105 bpm, and blood pressure in both arms was 110/60 mmHg. Rapid blood gas analysis revealed significant lactic acidosis (7 mmol/L). There were no signs of infection.

**Case 2.** A 38-year-old woman presented with acute onset (< 2 h) left hemiparesis and psychomotor slowing with preserved alertness (Glasgow Coma Scale [GCS] score of 15 points). Neurologic examination revealed left-sided upper motor neuron signs with brisk reflexes, positive Trömner and Babinski signs, and sustained ankle clonus. She reported no headache. She had no significant medical history or long-term medication.

**Case 3.** A 70-year-old woman was admitted after her partner noted an irregular sleep-related breathing pattern and she remained unresponsive to his attempts to wake her up in the early morning (approximately 4:00 am). She had last been seen well the previous evening. On presentation at 4:38 am, she was unconscious with a GCS score of 8 points and exhibited anisocoria (left > right), a skew deviation, and urinary incontinence. No motor phenomena suggestive of seizure activity were observed.

While acute stroke protocols typically target the rapid identification of lateralized lesions, clinical ‘red flags’ in Cases 1 and 2 broadened the differential diagnoses beyond single-territory arterial occlusion, prompting MRI as the first-line imaging modality. Conversely, in Case 3, the low GCS prompted emergency CT imaging to evaluate life-threatening differential diagnoses including intracranial hemorrhage, basilar artery occlusion, or acute intracranial hypertension. Across all three cases, imaging revealed bilateral, symmetric lesions resulting from distinct vascular mechanisms.

In Case 1 (Fig. 1, top row), MRI demonstrated bilateral frontoparietal watershed infarctions. This distribution, alongside elevated serum lactate, suggested systemic hypoperfusion. Thoracic CT angiography (CTA) confirmed an acute aortic dissection as underlying pathology. Thrombolysis was withheld due to the risk of fatal hemorrhage. The patient underwent immediate surgical management. After clinical stabilization, he was transferred to rehabilitation.

**Fig. 1:**
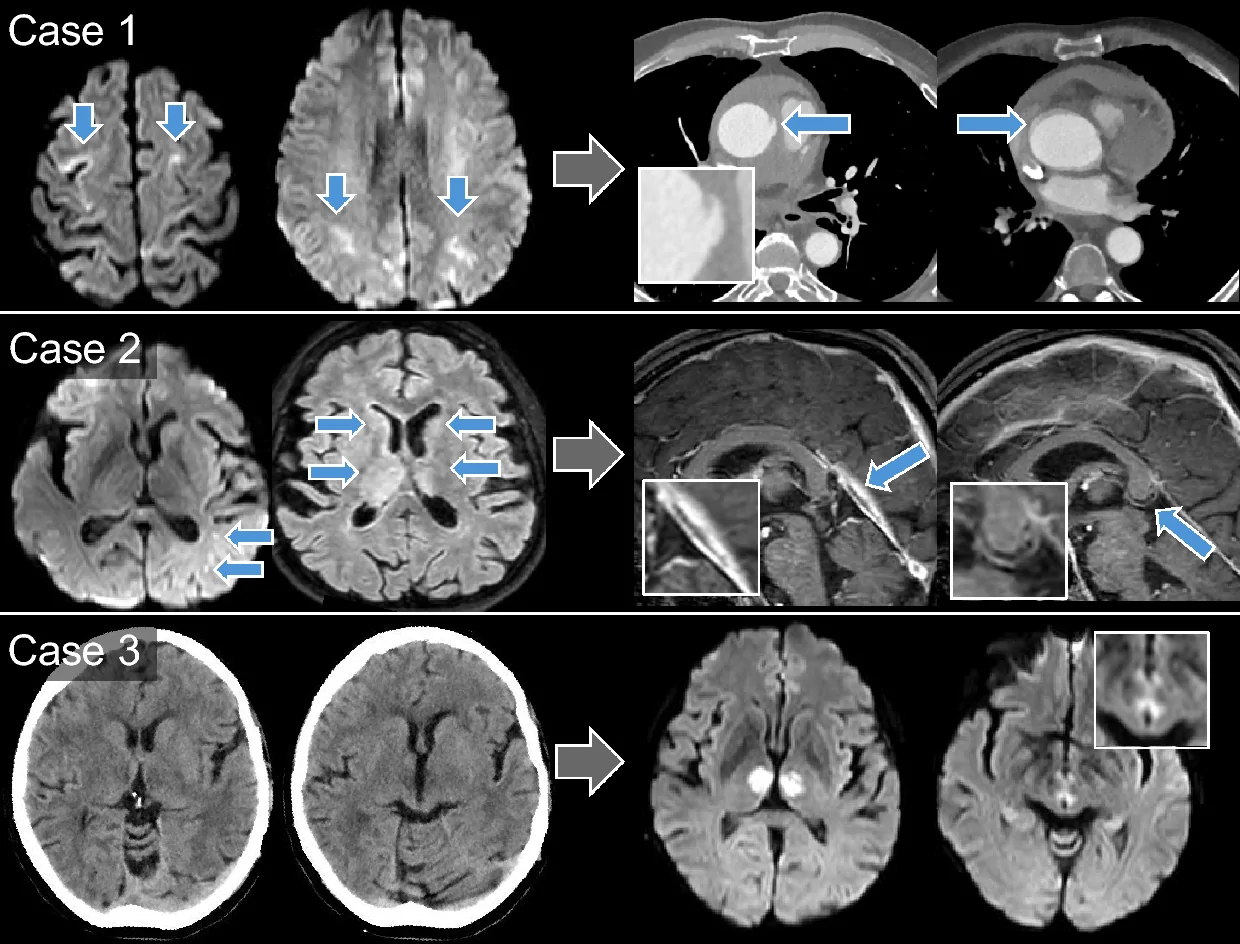
Initial neuroimaging findings (left) and additional diagnostic imaging (right). In Case 1 (top row), diffusion-weighted MR imaging (DWI, left) demonstrated bilateral frontoparietal watershed infarctions. Subsequent thoracic CTA (right) demonstrated aortic dissection. In Case 2 (middle row), DWI and FLAIR imaging (left) showed small parieto-occipital ischemic lesions without a clinical correlate and bilateral thalamostriatal edema. MR venography (right) showed thrombosis of the straight sinus and vein of Rosenthal. In Case 3 (bottom row), non-contrast CT (left) showed symmetric bilateral thalamic hypodensities extending toward the mesencephalon as well as an older lacunar right-hemispheric defect. MRI (DWI, right) demonstrated AOP infarction with bilateral thalamic and mesencephalic involvement

In Case 2 (Fig. 1, middle row), MRI showed non-territorial diffusion restriction and symmetric bilateral thalamostriatal edema, raising suspicion for venous congestion. MR venography confirmed deep cerebral venous thrombosis. Shortly after admission and before treatment, the patient developed left facial paresis and spastic tetraparesis with bilateral dystonic hand postures ("thalamic hand"). Marked clinical improvement was observed under therapeutic anticoagulation. The patient was discharged with mild residual facial paresis (modified Rankin Scale: 1).

In Case 3 (Fig. 1, bottom row), non-contrast CT identified symmetric thalamic hypodensities and an older right-hemispheric lacunar defect. Follow-up MRI confirmed an artery of Percheron (AOP) infarction extending to the mesencephalon [1]. These bilateral thalamic and mesencephalic lesions reflected the patient's impaired consciousness and oculomotor signs. Recanalization was withheld as initial CT hypodensities indicated established infarction. The patient transitioned to rehabilitation with residual disorientation, memory impairment, and fluctuating alertness. A vertebral artery stenosis was considered as a potential embolic source. Secondary prevention was initiated with antiplatelet and statin therapy.

Acute, unilateral neurologic deficits are among the clinical hallmarks of stroke, and emergency protocols are optimized for the rapid detection of single-territory infarctions. The interpretation of neuroimaging findings in this setting relies on the principle of asymmetry, comparing the lesional with the contralesional hemisphere. This principle is not only embedded in clinical practice but also reflected in widely used scoring systems such as ASPECTS, which quantifies ischemic changes by detecting interhemispheric differences [2]. Automated diagnostic tools, including AI-assisted platforms, frequently depend on contralateral comparison to identify and characterize imaging findings [3–6]. In this context, symmetry exerts a dual influence on clinical reasoning: it can reduce diagnostic accuracy by under-recognition of subtle bilateral pathologies (as for the ischemia in Case 3), or it can serve as a ‘red flag’ that the underlying mechanism may deviate from single-territory arterial occlusion (as in Case 1 and the FLAIR findings in Case 2).

The expectation that stroke-related imaging findings are mostly asymmetrical is grounded in vascular neuroanatomy: The cerebral arterial supply is organized as paired vascular territories, and, with the exception of the basilar system, single-vessel occlusion typically produces unilateral infarction. Consequently, bilateral symmetric brain lesions are often attributed to non-vascular etiologies. For example, a wide range of metabolic, toxic, infectious, and inflammatory processes preferentially affect the gray matter nuclei and the cortex in symmetric distributions, including carbon monoxide poisoning, osmotic demyelination, Wernicke encephalopathy, Wilson disease, autoimmune encephalitis, and acute necrotizing encephalopathy [7]. These differential diagnoses are well established and should be routinely considered when symmetric pathology is present.

However, especially in the setting of hyperacute neurologic deficits, several vascular mechanisms may violate the rule of asymmetry (Table 1). These range from systemic conditions causing diffuse vascular disease, such as cerebral vasculitis and hyperviscosity syndromes, to the following mechanisms, which are particularly relevant in the acute setting and must be recognized to avoid management errors.

**Table 1:** Examples of vascular and hemodynamic mechanisms causing bilateral symmetric imaging findings

Mechanism		Imaging pattern
*Diffuse vasculopathy/hyperviscosity*		
	Vessel wall: cerebral vasculitis (primary or secondary, e.g., SLE)	Bilateral multifocal infarcts across territories, often of different ages; hyperviscosity also watershed infarcts or venous thrombosis
	Blood: hyperviscosity, e.g., cellular (SCD, leukemic blast crisis, PV, ET) or plasma (MM, Waldenström macroglobulinemia, cryoglobulinemia, severe hyperlipidemia)	
*Hemodynamic failure / Global hypoxia*		
	Proximal arterial: aortic arch or bilateral carotid stenosis (e.g., atherosclerosis, Takayasu arteritis, radiation vasculopathy); aortic dissection *(Case 1)*	Bilateral watershed infarction; with severe or prolonged failure, diffuse cortical and deep gray matter injury
	Cardiac: heart failure, cardiac arrest	
	Systemic: sepsis, hypotension, anemia	
*Vascular midline obstruction*		
	Venous: intracranial: vein of Galen, straight sinus *(Case 2),* sagittal sinuses; extracranial: superior vena cava	Bilateral subcortical congestion and edema; with extracranial obstruction: diffuse cerebral edema
	Arterial: top-of-the-basilar occlusion	Bilateral PCA, thalamic and brainstem infarction
*Midline arterial variants*		
	A. of Percheron *(Case 3),* azygos or bihemispheric ACA, median artery of the corpus callosum	AOP: bilateral paramedian thalamic ± rostral midbrain; ACA: bilateral medial frontal

Mechanisms are grouped by pathophysiological principle. The examples given illustrate each group rather than enumerating all reported causes. Cases from the present series are indicated in italics. *PCA* posterior cerebral artery, *ACA* anterior cerebral artery, *AOP* artery of Percheron, *SLE* systemic lupus erythematosus, *SCD* sickle cell disease, *PV* polycythemia vera, *ET* essential thrombocythemia, *MM* multiple myeloma

**Hemodynamic failure.** Global or systemic hypoperfusion preferentially affects the watershed zones between major arterial territories, whereas more prolonged or severe hypoperfusion leads to diffuse cortical and deep gray matter injury resembling hypoxic-ischemic encephalopathy. Potential causes (Table 1) include aortic dissection, which is painless in 7–18% and presents with neurologic symptoms in 25–35% of these cases [8]. Notably, neurologic deficits have been reported as the predominant symptom in painless aortic dissection [8]. In Case 1, psychomotor slowing and unexplained lactic acidosis were important red flags: recognition of a proximal aortic rather than a primary cerebral mechanism prevented thrombolysis, which may carry the risk of fatal hemorrhage in unrecognized aortic dissection [9]. Beyond acute aortic dissection, stenoocclusive disease of the aortic arch and supra-aortic vessels, such as Takayasu arteritis or radiation-induced vasculopathy after head-and-neck or mediastinal radiotherapy, may also cause bilateral hypoperfusion. However, these conditions less often present hyperacutely. Pulse or blood pressure differences between the arms and vascular bruits can be useful bedside clues.

**Venous obstruction.** Because the cerebral venous system converges on midline structures (Table 1), occlusion can produce bilateral congestion and edema [10, 11]. While cerebral venous thrombosis accounts for only 0.5–3% of all strokes [10], bilateral thalamic changes are seen in approximately 60% of cases where the deep venous system is affected [11]. The ensuing imaging pattern may closely mimic that of metabolic or inflammatory encephalopathies and requires dedicated venography to be detected [7, 10]. In Case 2, signs of corticospinal disinhibition, which are atypical for hyperacute arterial ischemia, redirected imaging toward MR venography. Therapeutic anticoagulation is the standard of care and stands in contrast to thrombolysis, which would otherwise have been considered for ischemic stroke [10]. Extracranial venous outflow obstruction, for example in superior vena cava syndrome, may similarly cause bilateral cerebral venous congestion. It may result from compression by an aortic aneurysm, mediastinal mass, or bulky lymphadenopathy, or from thrombosis related to central venous catheters, pacemaker leads, or tumor invasion. Facial and upper-extremity swelling, jugular venous distension, and visible thoracic collateral veins point to this mechanism and were absent in Case 2.

**Midline vascular variants.** Anatomical variants creating a single bottleneck arterial supply for bilateral structures are best exemplified by the AOP, a single microvascular thalamoperforating trunk projecting from the P1 segment that supplies both paramedian thalami and the rostral midbrain [1]. This vascular variant is estimated to be present in 4–12% of the population and accounts for 0.1–2% of ischemic strokes and 4–18% of thalamic strokes [1]. Notably, CT imaging is frequently nondiagnostic. The AOP is often invisible on standard CTA, and early symmetric thalamic hypodensities, if demarcated at all, often fall below the threshold for side-to-side detection [1]. In Case 3, reduced alertness disproportionate to the subtle CT findings was the red flag that ultimately prompted further workup. In contrast to Cases 1 and 2, Case 3 highlights the risk of missing the more subtle bilateral symmetric findings, thereby precluding the opportunity for acute recanalization or targeted secondary prevention.

These cases illustrate how, in the hyperacute setting, recognizing symmetry may directly influence diagnostic and therapeutic decisions. Although non-lateralizing deficits and bilateral symmetric lesions are often associated with non-vascular conditions, they should not lower the suspicion of acute cerebrovascular disease. Instead, lesion distribution and accompanying clinical findings should guide further vascular imaging (Table 1). Bilateral watershed infarctions combined with signs of systemic hypoperfusion, abnormal peripheral pulses, or unexplained lactic acidosis should raise suspicion for aortic dissection, in which intravenous thrombolysis may have fatal consequences [9]. Symmetric thalamic edema extending beyond arterial territories should prompt venous imaging to exclude deep cerebral venous thrombosis, for which therapeutic anticoagulation is indicated. Finally, bilateral paramedian thalamic lesions accompanied by impaired consciousness or oculomotor abnormalities should suggest artery of Percheron infarction, warranting timely assessment for acute reperfusion treatment.

Importantly, these patterns challenge the conventional emphasis on interhemispheric asymmetry in stroke imaging. Although side-to-side comparison is a useful diagnostic principle, it can foster a systematic blind spot for the vascular mechanisms described here. Automated tools relying on contralateral comparison share this vulnerability, albeit to varying degrees. Tools operating on diffusion-weighted imaging, where lesions are identifiable by prominent intensity alterations and commonly serve as the reference standard [12], may be less dependent on interhemispheric comparisons. By contrast, where ischemic change is subtle, side-to-side comparison carries substantial diagnostic information and both non-contrast CT [5] and CT angiography-based [6] analyses have been reported to perform better when contralateral information is included. For example, automated perfusion analyses use the contralateral hemisphere as their denominator, defining the ischemic core as cerebral blood flow below 30% of the opposite side [13]. Such relative measures assume an unaffected reference side, a condition not met when hypoperfusion is bilateral. Midline vascular structures and variants pose a further challenge, as they lack a contralateral anatomical counterpart.

As automated imaging tools become integral to acute stroke workflows [14], clinicians must remain alert to vascular abnormalities that may escape asymmetry-based detection [15]. This series of patients with bihemispheric stroke in the hyperacute setting illustrates a shared diagnostic challenge across distinct vascular mechanisms: bilateral, approximately symmetric lesions may require clinicians to move beyond asymmetry-based reasoning and standard diagnostic algorithms developed primarily for unilateral stroke. Recognizing signs of bilateral stroke may be crucial to identifying vascular emergencies with fundamentally different treatment strategies.

## Data Availability

Data sharing is not applicable to this article as no datasets were generated or analyzed during the current study.
